# Bis[μ-1-(3,5-di­chloro­pyridin-2-yl)-2-(pyridin-3-yl­methyl­idene)hydrazine]bis­[(nitrato-κ*O*)silver(I)] aceto­nitrile disolvate

**DOI:** 10.1107/S1600536813024021

**Published:** 2013-09-04

**Authors:** Ying Guo, Hua Cai

**Affiliations:** a College of Science, Civil Aviation University of China, Tianjin 300300, People’s Repulic of China

## Abstract

In the centrosymmetric binuclear title complex, [Ag_2_(NO_3_)_2_(C_11_H_8_Cl_2_N_4_)_2_]·2CH_3_CN, the Ag^I^ atom is four-coordinated and exhibits a highly distorted tetrahedral coordination sphere defined by three N atoms from two 1-(3,5-di­chloro­pyridin-2-yl)-2-(pyridin-3-yl­methyl­idene)hy­drazine ligands and one O atom from a nitrate anion. Inter­molecular N—H⋯O hydrogen bonds link the complex mol­ecules, resulting in a two-dimensional supra­molecular structure parallel to (001).

## Related literature

For background to compounds with metal–organic framework structures, see: Barnett & Champness (2003 ▶); Roesky & Andruh (2003 ▶); Zaworotko (2000 ▶).
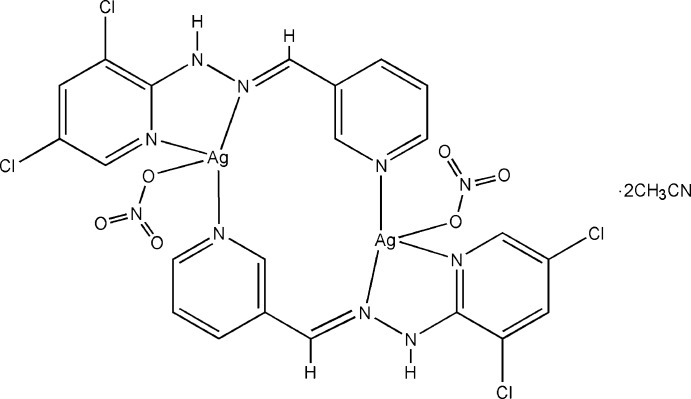


## Experimental

### 

#### Crystal data

[Ag_2_(NO_3_)_2_(C_11_H_8_Cl_2_N_4_)_2_]·2C_2_H_3_N
*M* *_r_* = 956.10
Orthorhombic, 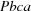

*a* = 15.3862 (19) Å
*b* = 8.2397 (10) Å
*c* = 27.198 (4) Å
*V* = 3448.1 (8) Å^3^
*Z* = 4
Mo *K*α radiation
μ = 1.51 mm^−1^
*T* = 296 K
0.32 × 0.28 × 0.22 mm

#### Data collection

Bruker APEXII CCD diffractometer
Absorption correction: multi-scan (*SADABS*; Sheldrick, 1996 ▶) *T*_min_ = 0.645, *T*_max_ = 0.733
16325 measured reflections
3041 independent reflections
2498 reflections with *I* > 2σ(*I*)
*R*_int_ = 0.029

#### Refinement

*R*[*F* ^2^ > 2σ(*F* ^2^)] = 0.039
*wR*(*F* ^2^) = 0.113
*S* = 1.08
3041 reflections
227 parameters
H-atom parameters constrained
Δρ_max_ = 0.66 e Å^−3^
Δρ_min_ = −0.50 e Å^−3^

### 

Data collection: *APEX2* (Bruker, 2007 ▶); cell refinement: *SAINT* (Bruker, 2007 ▶); data reduction: *SAINT*; program(s) used to solve structure: *SHELXS97* (Sheldrick, 2008 ▶); program(s) used to refine structure: *SHELXL97* (Sheldrick, 2008 ▶); molecular graphics: *XP* in *SHELXTL* (Sheldrick, 2008 ▶) and *DIAMOND* (Brandenburg & Berndt, 1999 ▶); software used to prepare material for publication: *SHELXTL*.

## Supplementary Material

Crystal structure: contains datablock(s) lxl, I. DOI: 10.1107/S1600536813024021/hy2636sup1.cif

Structure factors: contains datablock(s) I. DOI: 10.1107/S1600536813024021/hy2636Isup2.hkl

Additional supplementary materials:  crystallographic information; 3D view; checkCIF report

## Figures and Tables

**Table 1:** Hydrogen-bond geometry (Å, °)

*D*—H⋯*A*	*D*—H	H⋯*A*	*D*⋯*A*	*D*—H⋯*A*
N2—H2⋯O3^i^	0.86	2.09	2.909 (5)	158

Symmetry code: (i) 
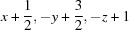
.

**Fig. 1.**
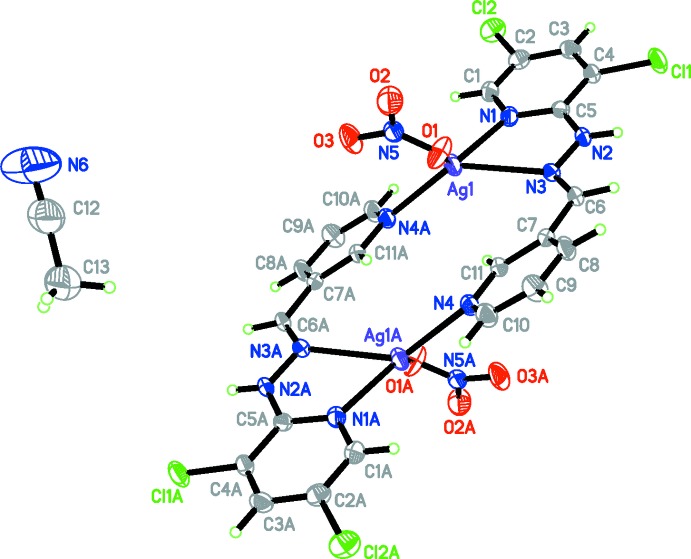
The molecular structure of the title compound. Displacement ellipsoids are shown at the 30% probability level. [Symmetry code: (A) 1-x, 2-y, 1-z.]

**Fig. 2.**
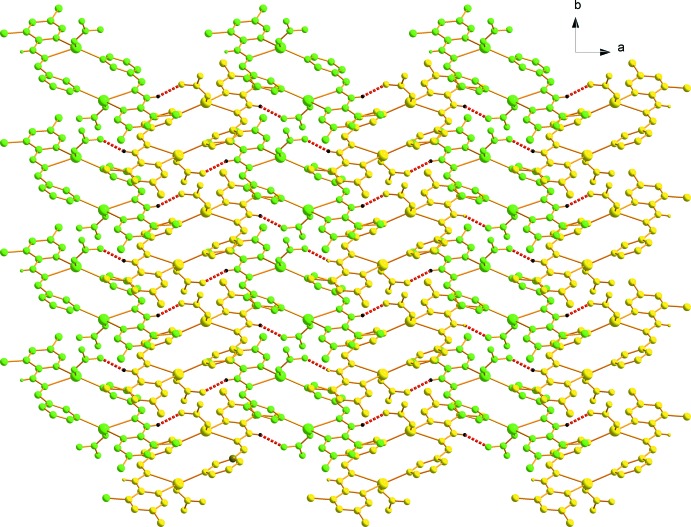
The two-dimensional supramolecular structure of the title compound, showing N—H···O hydrogen bonds as red dashed lines.

## supplementary crystallographic information

### 1. Comment

Neutral organic ligands containing rigid or flexible spacers, such as 4,4'-bipyridine, 1,2-bis(4-pyridyl)ethane, 1,2-bis(4-pyridyl)propane and many others, have been used to generate a rich variety of metal-organic architectures with different metal ions by various reaction procedure (Barnett & Champness, 2003; Roesky & Andruh, 2003; Zaworotko, 2000). In our recent research, we have initiated a synthetic approach employing 1-(3,5-dichloropyridin-2-yl)-2-(pyridin-3-ylmethylidene)hydrazine (*L*) upon reaction with differnent metal ions to construct new functional frameworks. To explore this series, we synthesized the title compound, a new Ag(I) complex based on the *L* ligand.

In the title complex (Fig. 1), the Ag^I^ atom is four-coordinated and exhibits a highly distorted tetrahedral geometry defined by three N atoms from two *L* ligands and one O from a nitrate anion. The *L* ligands bridge two Ag^I^ atoms, resulting in a centrosymmetric binuclear unit. The 2-pyridyl and 3-pyridyl rings in the ligand are not coplanar, with a dihedral angle of 25.74 (16)°. Intermolecular N—H···O hydrogen bonds (Table 1) extend the binuclear units into a two-dimensional supramolecular structure parallel to (001), as shown in Fig. 2.

### 2. Experimental

AgNO_3_ (17.0 mg, 0.1 mmol) and 1-(3,5-dichloropyridin-2-yl)-2-(pyridin-3-ylmethyl)diazene (22.2 mg, 0.1 mmol) were mixed in a CH_3_CN/H_2_O (20 ml, 1:1 *v*/*v*) solution with vigorous stirring for *ca* 30 min. The resulting solution was filtered and left to stand at room temperature. Colorless block crystals of the title compound suitable for X-ray analysis were obtained in 85% yield by slow evaporation of the solvent over a period of 1 week. Analysis, calculated for C_26_H_22_Ag_2_Cl_4_N_12_O_6_: C 50.42, H 5.14, N 8.40%; found: C 50.45, H 5.03, N 8.32%.

### 3. Refinement

Although all H atoms were visible in difference maps, they were finally placed in geometrically calculated positions and refined as riding atoms, with C—H = 0.93 (aromatic), 0.96 (methyl) and N—H = 0.86 Å and with *U*_iso_(H) = 1.2(1.5 for methyl)*U*_eq_(C, N).

### Crystal data

**Table:**

[Ag_2_(NO_3_)_2_(C_11_H_8_Cl_2_N_4_)_2_]·2C_2_H_3_N	*F*(000) = 1888
*M**_r_* = 956.10	*D*_x_ = 1.842 Mg m^−^^3^
Orthorhombic, *P**b**c**a*	Mo *K*α radiation, λ = 0.71073 Å
Hall symbol: -P 2ac 2ab	Cell parameters from 6506 reflections
*a* = 15.3862 (19) Å	θ = 2.7–29.1°
*b* = 8.2397 (10) Å	µ = 1.51 mm^−^^1^
*c* = 27.198 (4) Å	*T* = 296 K
*V* = 3448.1 (8) Å^3^	Block, colorless
*Z* = 4	0.32 × 0.28 × 0.22 mm

### Data collection

**Table:**

Bruker APEXII CCD diffractometer	3041 independent reflections
Radiation source: fine-focus sealed tube	2498 reflections with *I* > 2σ(*I*)
Graphite monochromator	*R*_int_ = 0.029
φ and ω scans	θ_max_ = 25.0°, θ_min_ = 1.5°
Absorption correction: multi-scan (*SADABS*; Sheldrick, 1996)	*h* = −15→18
*T*_min_ = 0.645, *T*_max_ = 0.733	*k* = −6→9
16325 measured reflections	*l* = −32→26

### Refinement

**Table:**

Refinement on *F*^2^	Primary atom site location: structure-invariant direct methods
Least-squares matrix: full	Secondary atom site location: difference Fourier map
*R*[*F*^2^ > 2σ(*F*^2^)] = 0.039	Hydrogen site location: inferred from neighbouring sites
*wR*(*F*^2^) = 0.113	H-atom parameters constrained
*S* = 1.08	*w* = 1/[σ^2^(*F*_o_^2^) + (0.038*P*)^2^ + 14.4907*P*] where *P* = (*F*_o_^2^ + 2*F*_c_^2^)/3
3041 reflections	(Δ/σ)_max_ = 0.001
227 parameters	Δρ_max_ = 0.66 e Å^−^^3^
0 restraints	Δρ_min_ = −0.50 e Å^−^^3^

### Special details

**Table:**

Geometry. All e.s.d.'s (except the e.s.d. in the dihedral angle between two l.s. planes) are estimated using the full covariance matrix. The cell e.s.d.'s are taken into account individually in the estimation of e.s.d.'s in distances, angles and torsion angles; correlations between e.s.d.'s in cell parameters are only used when they are defined by crystal symmetry. An approximate (isotropic) treatment of cell e.s.d.'s is used for estimating e.s.d.'s involving l.s. planes.
Refinement. Refinement of *F*^2^ against ALL reflections. The weighted *R*-factor *wR* and goodness of fit *S* are based on *F*^2^, conventional *R*-factors *R* are based on *F*, with *F* set to zero for negative *F*^2^. The threshold expression of *F*^2^ > σ(*F*^2^) is used only for calculating *R*-factors(gt) *etc*. and is not relevant to the choice of reflections for refinement. *R*-factors based on *F*^2^ are statistically about twice as large as those based on *F*, and *R*- factors based on ALL data will be even larger.

### Fractional atomic coordinates and isotropic or equivalent isotropic displacement parameters (Å^2^)

**Table:**

	*x*	*y*	*z*	*U*_iso_*/*U*_eq_	
Ag1	0.56792 (2)	0.75776 (5)	0.548365 (16)	0.04728 (16)	
Cl1	0.91981 (8)	0.6283 (2)	0.62236 (6)	0.0593 (4)	
Cl2	0.65242 (12)	0.4004 (2)	0.73037 (5)	0.0635 (4)	
O1	0.5766 (3)	0.6995 (6)	0.45874 (17)	0.0733 (14)	
O2	0.5395 (3)	0.5169 (6)	0.40845 (17)	0.0692 (12)	
O3	0.4486 (2)	0.5938 (6)	0.46331 (16)	0.0665 (13)	
N1	0.6643 (3)	0.6706 (5)	0.60951 (15)	0.0399 (10)	
N2	0.7784 (2)	0.7820 (5)	0.56417 (15)	0.0362 (9)	
H2	0.8333	0.7953	0.5598	0.043*	
N3	0.7204 (2)	0.8532 (5)	0.53260 (14)	0.0342 (9)	
N4	0.5645 (2)	1.1349 (5)	0.43586 (15)	0.0384 (9)	
N5	0.5217 (3)	0.6047 (5)	0.44344 (16)	0.0419 (10)	
N6	0.1005 (11)	0.4204 (14)	0.2093 (4)	0.186 (6)	
C1	0.6359 (3)	0.5835 (6)	0.6484 (2)	0.0445 (12)	
H1	0.5764	0.5707	0.6530	0.053*	
C2	0.6916 (4)	0.5135 (7)	0.68122 (19)	0.0444 (12)	
C3	0.7808 (4)	0.5264 (6)	0.67384 (19)	0.0445 (12)	
H3	0.8198	0.4768	0.6952	0.053*	
C4	0.8098 (3)	0.6139 (6)	0.63428 (19)	0.0394 (11)	
C5	0.7495 (3)	0.6900 (6)	0.60256 (17)	0.0341 (10)	
C6	0.7533 (3)	0.9297 (6)	0.49620 (17)	0.0352 (10)	
H6	0.8134	0.9331	0.4929	0.042*	
C7	0.6992 (3)	1.0122 (6)	0.45957 (17)	0.0332 (10)	
C8	0.7328 (3)	1.0400 (7)	0.41291 (19)	0.0455 (13)	
H8	0.7896	1.0107	0.4053	0.055*	
C9	0.6801 (4)	1.1120 (8)	0.3781 (2)	0.0531 (14)	
H9	0.7006	1.1303	0.3464	0.064*	
C10	0.5973 (3)	1.1562 (7)	0.3907 (2)	0.0478 (13)	
H10	0.5622	1.2032	0.3668	0.057*	
C11	0.6150 (3)	1.0632 (6)	0.46957 (18)	0.0344 (10)	
H11	0.5928	1.0468	0.5010	0.041*	
C12	0.1048 (8)	0.5540 (14)	0.2028 (3)	0.107 (3)	
C13	0.1061 (8)	0.7257 (12)	0.1945 (5)	0.134 (4)	
H13A	0.1004	0.7470	0.1599	0.201*	
H13B	0.1600	0.7698	0.2061	0.201*	
H13C	0.0586	0.7755	0.2117	0.201*	

### Atomic displacement parameters (Å^2^)

**Table:**

	*U*^11^	*U*^22^	*U*^33^	*U*^12^	*U*^13^	*U*^23^
Ag1	0.0295 (2)	0.0536 (3)	0.0587 (3)	0.00328 (18)	−0.00426 (17)	0.0089 (2)
Cl1	0.0305 (6)	0.0752 (10)	0.0723 (10)	0.0088 (7)	−0.0074 (6)	0.0185 (8)
Cl2	0.0796 (11)	0.0639 (10)	0.0469 (8)	−0.0064 (8)	0.0057 (7)	0.0126 (7)
O1	0.072 (3)	0.073 (3)	0.075 (3)	−0.039 (3)	0.007 (2)	−0.017 (2)
O2	0.057 (3)	0.073 (3)	0.078 (3)	−0.001 (2)	0.003 (2)	−0.024 (3)
O3	0.034 (2)	0.095 (4)	0.070 (3)	0.008 (2)	0.0116 (19)	0.024 (3)
N1	0.032 (2)	0.042 (2)	0.045 (2)	−0.0037 (18)	−0.0038 (18)	0.006 (2)
N2	0.0230 (19)	0.038 (2)	0.047 (2)	0.0018 (16)	−0.0047 (17)	0.0053 (18)
N3	0.029 (2)	0.033 (2)	0.040 (2)	0.0026 (17)	−0.0061 (17)	0.0009 (18)
N4	0.030 (2)	0.042 (2)	0.042 (2)	0.0005 (18)	−0.0047 (17)	0.0069 (19)
N5	0.035 (2)	0.042 (2)	0.049 (3)	0.001 (2)	−0.0004 (19)	0.006 (2)
N6	0.354 (18)	0.105 (7)	0.099 (7)	0.039 (10)	−0.090 (9)	−0.002 (6)
C1	0.035 (3)	0.045 (3)	0.054 (3)	−0.004 (2)	0.003 (2)	0.005 (3)
C2	0.055 (3)	0.041 (3)	0.037 (3)	−0.003 (3)	0.002 (2)	0.003 (2)
C3	0.050 (3)	0.041 (3)	0.042 (3)	0.006 (2)	−0.011 (2)	0.004 (2)
C4	0.029 (2)	0.041 (3)	0.048 (3)	0.002 (2)	−0.006 (2)	−0.002 (2)
C5	0.035 (2)	0.029 (2)	0.038 (2)	0.002 (2)	−0.007 (2)	−0.002 (2)
C6	0.023 (2)	0.037 (3)	0.045 (3)	0.0032 (19)	−0.003 (2)	0.000 (2)
C7	0.028 (2)	0.029 (2)	0.042 (3)	−0.0029 (19)	−0.0025 (19)	0.003 (2)
C8	0.026 (3)	0.056 (3)	0.054 (3)	0.005 (2)	0.005 (2)	0.006 (3)
C9	0.051 (3)	0.070 (4)	0.039 (3)	0.008 (3)	0.007 (2)	0.012 (3)
C10	0.041 (3)	0.056 (3)	0.046 (3)	0.003 (3)	−0.009 (2)	0.010 (3)
C11	0.026 (2)	0.035 (3)	0.041 (3)	0.0004 (19)	0.000 (2)	0.002 (2)
C12	0.134 (9)	0.095 (7)	0.091 (6)	0.016 (7)	−0.054 (6)	−0.013 (6)
C13	0.123 (9)	0.090 (8)	0.189 (12)	0.015 (6)	−0.057 (8)	−0.022 (7)

### Geometric parameters (Å, º)

**Table:**

Ag1—N4^i^	2.262 (4)	C1—H1	0.9300
Ag1—N1	2.341 (4)	C2—C3	1.391 (8)
Ag1—O1	2.488 (5)	C3—C4	1.370 (7)
Ag1—N3	2.511 (4)	C3—H3	0.9300
Cl1—C4	1.727 (5)	C4—C5	1.415 (7)
Cl2—C2	1.738 (5)	C6—C7	1.465 (6)
O1—N5	1.224 (6)	C6—H6	0.9300
O2—N5	1.227 (6)	C7—C8	1.389 (7)
O3—N5	1.251 (6)	C7—C11	1.389 (6)
N1—C5	1.334 (6)	C8—C9	1.381 (7)
N1—C1	1.352 (6)	C8—H8	0.9300
N2—C5	1.365 (6)	C9—C10	1.368 (8)
N2—N3	1.370 (5)	C9—H9	0.9300
N2—H2	0.8600	C10—H10	0.9300
N3—C6	1.278 (6)	C11—H11	0.9300
N4—C10	1.339 (7)	C12—C13	1.433 (14)
N4—C11	1.339 (6)	C13—H13A	0.9600
N4—Ag1^i^	2.262 (4)	C13—H13B	0.9600
N6—C12	1.117 (13)	C13—H13C	0.9600
C1—C2	1.365 (7)		
			
N4^i^—Ag1—N1	123.76 (15)	C3—C4—C5	119.9 (5)
N4^i^—Ag1—O1	108.04 (16)	C3—C4—Cl1	120.2 (4)
N1—Ag1—O1	127.07 (17)	C5—C4—Cl1	119.9 (4)
N4^i^—Ag1—N3	138.70 (14)	N1—C5—N2	119.7 (4)
N1—Ag1—N3	68.01 (13)	N1—C5—C4	120.4 (4)
O1—Ag1—N3	80.96 (13)	N2—C5—C4	119.9 (4)
N5—O1—Ag1	114.8 (3)	N3—C6—C7	122.1 (4)
C5—N1—C1	119.5 (4)	N3—C6—H6	118.9
C5—N1—Ag1	119.0 (3)	C7—C6—H6	118.9
C1—N1—Ag1	120.9 (3)	C8—C7—C11	118.4 (4)
C5—N2—N3	120.3 (4)	C8—C7—C6	119.1 (4)
C5—N2—H2	119.8	C11—C7—C6	122.4 (4)
N3—N2—H2	119.8	C9—C8—C7	118.6 (5)
C6—N3—N2	116.1 (4)	C9—C8—H8	120.7
C6—N3—Ag1	131.0 (3)	C7—C8—H8	120.7
N2—N3—Ag1	111.5 (3)	C10—C9—C8	119.3 (5)
C10—N4—C11	117.8 (4)	C10—C9—H9	120.4
C10—N4—Ag1^i^	117.5 (3)	C8—C9—H9	120.4
C11—N4—Ag1^i^	124.4 (3)	N4—C10—C9	123.1 (5)
O1—N5—O2	119.1 (5)	N4—C10—H10	118.4
O1—N5—O3	121.3 (5)	C9—C10—H10	118.4
O2—N5—O3	119.6 (5)	N4—C11—C7	122.7 (5)
N1—C1—C2	122.2 (5)	N4—C11—H11	118.6
N1—C1—H1	118.9	C7—C11—H11	118.6
C2—C1—H1	118.9	N6—C12—C13	177.4 (16)
C1—C2—C3	119.6 (5)	C12—C13—H13A	109.5
C1—C2—Cl2	120.7 (4)	C12—C13—H13B	109.5
C3—C2—Cl2	119.6 (4)	H13A—C13—H13B	109.5
C4—C3—C2	118.4 (5)	C12—C13—H13C	109.5
C4—C3—H3	120.8	H13A—C13—H13C	109.5
C2—C3—H3	120.8	H13B—C13—H13C	109.5
			
N4^i^—Ag1—O1—N5	−59.5 (5)	C2—C3—C4—Cl1	−178.0 (4)
N1—Ag1—O1—N5	108.6 (4)	C1—N1—C5—N2	−178.0 (4)
N3—Ag1—O1—N5	162.0 (4)	Ag1—N1—C5—N2	10.3 (6)
N4^i^—Ag1—N1—C5	−145.0 (3)	C1—N1—C5—C4	2.9 (7)
O1—Ag1—N1—C5	48.7 (4)	Ag1—N1—C5—C4	−168.8 (3)
N3—Ag1—N1—C5	−10.2 (3)	N3—N2—C5—N1	−0.5 (7)
N4^i^—Ag1—N1—C1	43.4 (4)	N3—N2—C5—C4	178.5 (4)
O1—Ag1—N1—C1	−122.9 (4)	C3—C4—C5—N1	−3.3 (7)
N3—Ag1—N1—C1	178.2 (4)	Cl1—C4—C5—N1	175.5 (4)
C5—N2—N3—C6	−176.3 (4)	C3—C4—C5—N2	177.6 (5)
C5—N2—N3—Ag1	−8.3 (5)	Cl1—C4—C5—N2	−3.6 (7)
N4^i^—Ag1—N3—C6	−68.5 (5)	N2—N3—C6—C7	−179.8 (4)
N1—Ag1—N3—C6	174.9 (5)	Ag1—N3—C6—C7	15.0 (7)
O1—Ag1—N3—C6	38.6 (4)	N3—C6—C7—C8	−156.3 (5)
N4^i^—Ag1—N3—N2	125.8 (3)	N3—C6—C7—C11	23.3 (7)
N1—Ag1—N3—N2	9.2 (3)	C11—C7—C8—C9	−2.1 (8)
O1—Ag1—N3—N2	−127.1 (3)	C6—C7—C8—C9	177.4 (5)
Ag1—O1—N5—O2	−149.3 (4)	C7—C8—C9—C10	1.1 (9)
Ag1—O1—N5—O3	29.4 (7)	C11—N4—C10—C9	−1.7 (8)
C5—N1—C1—C2	−0.1 (8)	Ag1^i^—N4—C10—C9	173.1 (5)
Ag1—N1—C1—C2	171.5 (4)	C8—C9—C10—N4	0.9 (10)
N1—C1—C2—C3	−2.4 (8)	C10—N4—C11—C7	0.6 (7)
N1—C1—C2—Cl2	−179.2 (4)	Ag1^i^—N4—C11—C7	−173.8 (3)
C1—C2—C3—C4	2.0 (8)	C8—C7—C11—N4	1.3 (7)
Cl2—C2—C3—C4	178.8 (4)	C6—C7—C11—N4	−178.2 (4)
C2—C3—C4—C5	0.8 (8)		

Symmetry code: (i) −*x*+1, −*y*+2, −*z*+1.

### Hydrogen-bond geometry (Å, º)

**Table:**

*D*—H···*A*	*D*—H	H···*A*	*D*···*A*	*D*—H···*A*
N2—H2···O3^ii^	0.86	2.09	2.909 (5)	158

Symmetry code: (ii) *x*+1/2, −*y*+3/2, −*z*+1.
